# Comment on Bar-Sela et al. Cannabis Consumption Used by Cancer Patients During Immunotherapy Correlates with Poor Clinical Outcome. *Cancers* 2020, *12*, 2447

**DOI:** 10.3390/cancers17172754

**Published:** 2025-08-23

**Authors:** Brian J. Piper, Duncan X. Dobbins, Jason Graham, Thomas M. Churilla, Michael Bordonaro

**Affiliations:** 1 Department of Medical Education, Geisinger College of Health Sciences, Scranton, PA 18509, USAmbordonaro1@geisinger.edu (M.B.); 2 Center for Pharmacy Innovation & Outcomes, Geisinger College of Health Sciences, Danville, PA 17821, USA; ddobbins@geisinger.edu; 3 Department of Mathematics, University of Scranton, Scranton, PA 18510, USA; jason.graham@scranton.edu; 4 Northeast Radiation Oncology Centers, Dunmore, PA 18512, USA

**Keywords:** interaction, marijuana, nivolumab, oncology, pembrolizumab

## Abstract

The small (N = 102) prospective study by Bar-Sela and colleagues at Emek Medical Center in Israel) regarding diminished efficacy of immunotherapy in the setting of cannabis use would be an important discovery which could theoretically benefit the outcomes of oncology patients if verified by independent research teams, including by basic scientists. However, if this finding was spurious, clinical practice guidelines could recommend that oncology patients receiving immunotherapies be erroneously denied an evidence-based treatment for pain and chemotherapy-induced nausea and vomiting. Our full-length manuscript identified dozens of instances of unverifiable statistical information and even errors in arithmetic in this report. More briefly, our concerns regarding this well-cited (123 times) paper are as follows: (1) non-verifiable non-parametric statistics, including some that would change the statistical inferences; (2) difficulties with determining percentages; (3) switching from two-tailed tests in the Methods to one-tailed in the Results; (4) engaging in the unusual practice of floor rounding but not reporting this in the Methods; and (5) not reporting smoking, which could be a key confound. These concerns are serious errors that undermine the validity of the results and invalidate the conclusions that can be drawn from this prospective study about cannabis and immunotherapy.

## Simple Summary

Medical cannabis is an evidence-based treatment for chronic pain and chemotherapy-induced nausea and vomiting. Immunotherapies like pembrolizumab work by blocking proteins that stop the immune system from working properly. This commentary critically analyzes a small study by Bar-Sela and coworkers, which appeared to show that cannabis made immunotherapies work less well in patients. Scientific papers should describe what they did in the Methods section and then carefully adhere to this in the Results section. However, we found many instances where there were disagreements between the Methods and Results sections in Bar-Sela’s report. It is also riddled with errors in arithmetic, including unconventional rounding. The prior study did not consider the role of tobacco smoking, which may have been more important than cannabis use in their findings. We propose that artificial intelligence could be used by journals to identify mathematical and statistical errors and prevent studies that are not trustworthy from being published.

## 1. Introduction

The small (N = 102) prospective study by Bar-Sela and colleagues regarding a purported drug interaction between immunotherapy and cannabis [1] would be an important discovery which could theoretically benefit the outcomes of oncology patients, if verified by independent research teams, including basic scientists. The hypothetical interaction results in the immunotherapy being less effective and could indirectly result in poorer outcomes, including mortality. However, if this finding were spurious [2], clinical practice guidelines [3,4] could recommend that oncology patients receiving immunotherapies be erroneously denied an evidence-based treatment for pain and chemotherapy-induced nausea and vomiting [5]. Our full-length manuscript [6] identified dozens of instances of unverifiable statistical information, as well as errors in arithmetic in [1]. More briefly, our concerns regarding this well-cited (123 times) paper are as follows:

A. Table 1 and the associated text is the foundation of this report [1]. If this baseline information is inaccurate and the groups differ significantly on other variables besides cannabis, then it becomes very challenging to meaningfully assess the rest of this study. They reported (page 3, Section 2.1) that liver metastasis of the immunotherapy group (19% of N = 68 is 13) vs. the immunotherapy–cannabis group (67% of N = 34 is 23) had a *p* = 0.89. Using GraphPad Prism [7] and the two statistics listed in their Methods section (chi-square and Fisher’s exact tests) with the N provided, we were unable to verify this result. We determined that chi-square (1) = 23.375, *p* < 0.0001 and Fisher’s *p* < 0.0001.

Readers may also be confused by the numbers cited in the text for liver metastases (19% I-G, 67% IC-G) not matching what is in Table 1 (19.1% I-G, 32.3% IC-G). Nor do the *p* values match (*p* = 0.89 in text, and *p* = 0.2157 in the table).

B. Table 1 notes that 76% of the immunotherapy–cannabis group (76% of N = 34 is 26) were given immunotherapy as the second line of treatment versus 54% (54% of N = 68 is 37) of immunotherapy only, which had a *p*-value of “0.05178”. Again, we were unable to verify this with chi-square (1) = 4.67, *p* = 0.0317 and Fisher’s *p* = 0.0334.
C. Similarly, only one of the 22 *p*-values (4.5%) in the corrected Table 1 could be verified using the same statistics, as were reported in the Methods section [1]. Based on *p*-value agreement to at least three decimal places, the authors appear to have reported a different statistic (Yates) than was in their Methods section (chi-square or Fisher’s) on 18 of 22 (81.8%) of these statistics. This is a deviation from common scientific reporting conventions.
D. Although perhaps less impactful for their core conclusions, there appears to have been some difficulties in calculating percentages (i.e., a difference of 16.2% between the reported 22.0% and our calculated value of 38.2% for chronic diseases = 0 for cannabis users; a difference of 4.1% between the reported 34.1 and calculated 38.2% for high blood pressure for cannabis users; and a 10.3% difference between the reported 13.2% and the calculated 23.5% for bone metastasis for cannabis users) [6], which suggests an inattention to detail in the “corrected” Table 1. Further, the total percentages for chronic diseases for cannabis users (0 = 22.0%, 1 = 20.5%, ≥2 = 41.1%) adds up to 83.6% instead of the usual 100.0%.
E. The original authors also employed a type of “floor rounding” on 19 occasions [6]. For those who are unfamiliar (as we were), a floor function would take an input (e.g., 4.9) and convert it to the next lowest whole integer (4.0) [8]. As applied here, a number like 0.049999 if calculated to two decimal places would be listed as “0.04” with floor rounding. In retrospect, unusual practices like this should be noted in the Methods section.
F. The Methods section includes the standard statement that “We computed 2-tailed *p*-values, where *p* < 0.05 was considered a statistically significant result.” The sample size calculation sub-section reinforced the reader’s expectation of two-tailed statistical tests by stating “alpha of 5% for the two-tailed test.” for the complete response and partial response calculations. However, the Results section then notes that there was “(one-tailed *p* = 0.08) (Table 2).” [1]. Although we found [6,7] the one-tailed Fisher’s was “0.089”, which would commonly be reported as 0.09, this could be attributed to floor-rounding. The alkaline phosphatase level was reported as “0.09”, but we obtained a one-tailed chi-square of 0.0687, one-tailed Fischer’s of 0.1089, and even a one-tailed Yates of 0.1079. Switching the data analysis plan from the Methods to the Results section (i.e., from two-tailed to one-tailed) is incongruent with typical practice in scientific reporting. If the authors made their deidentified raw data and a detailed data dictionary publicly available on GitHub at https://github.com/ (assuming that this were acceptable to the Rambam IRB), this could allow for independent re-analysis of this and other statistics (Table 4, and Figure 3 and Figure 4).
G. Tobacco smoking is a defining theme of oncology that impacts cancer risk, diagnosis, and treatment [9,10]. This includes lung cancer, with a relative risk of seven among smokers [11]. Lung cancer patients that quit smoking may have a 20 to 35% improved survival [10]. Current smokers had a greater (Hazard Ratio = 1.48) likelihood of melanoma-associated death than non-smokers [12]. A meta-analysis revealed that current smokers with renal cell carcinoma had poorer progression-free survival (HR = 2.94) than non-smokers [13]. Note that about half the Bar-Sela sample from Israel consisted of non-small cell lung cancer, one-third melanoma, and the rest were renal cell carcinoma or other malignancies [1]. Among Israeli cancer survivors, significantly more cannabis users also used tobacco [14]. Adding further complexity, about one-third of cancer survivors with an active cannabis permit in Israel reported mixing tobacco with cannabis [14]. The Bar-Sela team should be commended for reporting smoking in Table 2 of their retrospective study [15] as this could be an important confound [9,10,11,12,13,14]. However, this key variable was never mentioned, and therefore, any variance due to this was not removed in their prospective study [1].
H. There is a very limited sample size within the cannabis group (n = 34), who were more likely to receive second line treatment, were more likely to have impaired performance statuses (ECOG ≥ 2) and had higher rates of baseline lymphopenia (all associated with worse clinical outcomes). Although none of these differences reach statistical significance, it does raise questions about important differences that may exist between the groups that may not have been apparent with this limited sample size. A median overall survival of six months seems unusually low in the modern immunotherapy era for these malignancies, which likely suggests additional unmeasured confounds associated with cannabis use and cancer outcomes.

## 2. Potential Solutions

We do not fault the reviewers, including the statistician reviewer (if there was one), for not identifying issues A–D. However, we do not believe it would be impossible for journals like *Cancers* to use software [16] or artificial intelligence to calculate percentages, chi-squares, and *t*-tests to assist the reviewers in flagging potential issues, particularly ones that change statistical decision making, earlier. Leveraging Large Language Models (LLMs) like OpenAI’s ChatGPT o1 model could appreciably reduce statistical errors in manuscripts, as demonstrated in Appendix A. With minimal input, such as copying relevant tables, specifying statistical tests, and simple prompt engineering, these models efficiently validate percentages, recalculate *p*-values, and flag discrepancies in parametric or non-parametric tests. The o1 model, highlighted in OpenAI’s report on learning to reason with LLMs [17], outperforms earlier models like 4o in statistical reasoning, often exhibiting the precision of a statistician by recognizing when tests like Fisher’s exact test were employed. Our conversation threads with 4o and o1 (Appendix A) demonstrate how o1 consistently identified errors and recalculated values that 4o missed, showcasing its ability to navigate complex statistical tasks with minimal guidance. This level of accuracy may not have been achievable with simpler models, but advanced LLMs like o1 offer valuable tools for both authors and reviewers, assisting peer review by automating statistical verification as a double-check and reducing the likelihood of publishing flawed findings.

Of course, AI models will need to be thoroughly tested and extensively evaluated to ensure their accuracy before being incorporated in the biomedical review process. Journals could begin this process by testing whether prompting authors to use AI to double-check the math of their preprint prior to submission would produce a quantifiable reduction in PubPeer comments or published corrections. Later, this could advance to more complicated tasks like identifying discrepancies between a reported correlation coefficient, or *t*-test, and the *p*-value when the degrees of freedom are provided. Repeating even simple (i.e., 2 × 2) non-parametric analyses would require greater standardization of how these are written than biomedical journals currently require. Authors could rebut erroneous AI feedback or make adjustments at the pre-submission stage. The utilization of LLMs preserves expert judgment for nuanced interpretation and contextual critique, while using AI for rapid, standardized checks that streamline the process for both journal staff and authors. AI can act as a first check for tedious math-checking, so reviewers can focus their mental energy on evaluating science.

Some reputable journals in psychology (*Psychological Science* and the *Journal of Experimental Social Psychology*) have incorporated the R application *statcheck* as a standard part of their peer review process [16]. The statistical standards, as well as transparency, of oncology should be at least as rigorous as those employed in the social sciences. Of course, human reviewers will continue to be crucial to thoroughly vetting methodologically suspect manuscripts, including identifying the omission of potentially key confounds (A, B, and G) prior to publication. If these are identified, then observational studies could use standard multivariate statistics to remove the variance attributable to them or, if the sample size was sufficient, stratify the sample accordingly (e.g., by nicotine use). Further, observational studies, with their potential for multiple unmeasured confounds, occasional reliance on self-reported data (e.g., smoking), and lack of random assignment, should not be interpreted in terms of causality. Controlled basic science investigations do not suffer from these limitations. A small (N = 7–9/group) mouse non-small cell lung cancer investigation revealed that survival was non-significantly longer in the anti-PD-1 antibody and tetrahydrocannabinol group (54 days) relative to the anti-PD-1 only group (31 days) [17].

More broadly, “cannabis” is not a single homogenous substance. One of the main cannabis producers in Israel, Tikun Olam Ltd., cultivates sixteen different strains, including ten chemotypes with more than 18% tetrahydrocannabinol and up to 1% cannabidiol, as well as two types with 1% tetrahydrocannabinol and ≥18% cannabidiol [18]. The US situation may be even more challenging, with almost two-thousand “strains” in an online database [19]. The impact of THC and CBD on the immune system is understudied [20]. More specifically, further research on whether checkpoint inhibitors differ in their potential interaction with certain cannabinoids is needed. Future efforts to examine the benefits, or harms, of cannabis would benefit from characterizing a single formulation with a known daily dose of the major cannabinioids with a single route of administration [21], which would facilitate the ability to draw meaningful inferences based on more defined pharmacokinetic and pharmacodynamic properties.

## 3. Conclusions

The authors of future narrative and systematic reviews, meta-analyses, and clinical practice guidelines may find ambiguous situations like these to be challenging. The writings of JPA Ioannidis may provide some guidance [2,22]. Issues A-C, F, and potentially G, appear to us to be serious errors that undermine the validity of the results and invalidate the conclusions. This commentary is not intended to question the integrity of Bar-Sela and colleagues. We leave it to others to infer whether A-G reflect typos, an inattention to detail, misinterpretation on our part or whether the underlying drug interaction is suspect. The retrospective [15] and prospective reports [1] have been criticized because cannabis use may only be a surrogate for a high burden of symptomatic disease [17]. Put more plainly, cannabis may be used as palliative care for those who recognize they are nearing the end of their lives. It also did not escape notice that one author of a recent review on phytocannabinoids as chemotherapy adjuncts [23] elected to omit the study in [15] from consideration due to statistical concerns. This may be a prudent approach. While writing from the perspective of an oncology researcher (MB), a pharmacist (DXD), an applied mathematician (JG), a radiation oncologist (TMC), and a cancer patient that has failed two immunotherapies (BJP), we would like to express our gratitude to Bar-Sela and colleagues for their thought-provoking manuscripts [1,15].

## Data Availability

All analyses were completed with the information publicly available in [1].

## Abbreviation

The following abbreviation is used in this manuscript:

LLM	Large Language Models

## Appendix A

Below, we present focused statistical audits of Bar-Sela et al. [1] using ChatGPT 4o and narrations of three threads. Key information is highlighted in green. Small discrepancies with rounding are highlighted in pink. See the exact threads in the pages below, with the corresponding numbers.

**1.**

https://chatgpt.com/share/677811e3-a490-800e-b7bc-fc6486a8098f (accessed on 4 January 2025)

- We gave the ChatGPT 4o model the paper and instructed it to go to Table 1 and perform a chi-square test for the gender- and number-matched samples. The results were good.
- Then, we instructed the model to now perform the same test for Table 1. Cont., with immunotherapy given as the first-line vs. second-line treatment. The 4o model produced a *p*-value of 0.0518, which matched that in the paper, likely due to bias. We found this to be inaccurate so we input the data into the o1 model (much better at statistics) and instructed it to perform the same task. See # 2.

**2.**

https://chatgpt.com/share/6778131d-ada0-800e-b3e5-ef57c388ef89 (accessed on 4 January 2025)

We instructed the model to perform the following task: “using the table below in brackets. calculate chi squared”. Then, we copy and pasted the portion of Table 1. Cont. that included immunotherapy.

- Because the o1 model does not read documents like 4o, it needs to be copy and pasted as text.
- This model found the *p*-value to be 0.03, not 0.0518 as the paper and 4o reported.
- https://openai.com/index/learning-to-reason-with-llms/ (accessed on 4 January 2025) shows how much more accurate o1 is, especially at math.

**3.**

https://chatgpt.com/share/677814bc-d30c-800e-9bef-0a1d9d2ad0ec (accessed on 4 January 2025)

- To see how o1 could perform on its own with little guidance, we said “below is a Table 1. Cont. for a paper. please recalculate values and check for accuracy”, and then, we copy and pasted the entire Table 1. Cont.
- The model found an error in the table where it marks the brain as the main site of metastasis, where Users (N = 34): 8/34 = 23.5%. The original table lists 13.2% for 8/34, which is incorrect.
- Then, with a very simple prompt, “thanks, now do chi squared please and thank you”, with very little guidance, it was able to distinguish the following:
  - Important Note
  - Many of the original *p*-values in your table appear to come from Fisher’s Exact Tests (common for smaller counts) or from combining multi-category variables into one test.
  - Chi--Square results on small or sparse counts (some cells <5) should be interpreted with caution.
- Thus, the *p*-values below will differ from the original table’s *p*-values if the original used Fisher’s Exact Test or a multi-level Chi--Square.
- Again, these will differ from your original table if those *p*-values were based on Fisher’s Exact Test or a multi-category test.
- This shows that intelligent models like o1 can be utilized to readily double-check statistics. It can easily navigate percentages and non-parametric tests with little context.

Copy and paste/screenshot of threads:

**1.**

**Me**:

*attached Gil Bar-Sela et al. “Cannabis Consumption Used by Cancer Patients

during Immunotherapy Correlates with Poor Clinical Outcome.” [1]

here’s a paper. Go to Table 1 and perform a chi-square test for the gender.

**ChatGPT 4o:**

The chi-square test for gender distribution between cannabis users and non-users resulted in:

• Chi-square value (χ^2^): 0.0057
  • *p*-value: 0.9399
  • Degrees of freedom (dof): 1
  • Expected frequencies:
○ Female: [21.33, 10.67]
○ Male: [46.67, 23.33]

The *p*-value of 0.94 indicates no statistically significant difference in gender distribution between the two groups.

Python, version 3.1

import scipy.stats as stats

import numpy as np

# Observed data from Table 1 for gender distribution

observed = np.array([[22, 10], # Female (Non-Users, Users)

[46, 24]]) # Male (Non-Users, Users)

# Perform Chi-Square Test

chi2, *p*, dof, expected = stats.chi2_contingency(observed)

chi2, *p*, dof, expected

**Me**:

now do the same thing for Table 1 cont: immunotherapy given as first line vs. second lined line

**ChatGPT 4o:**

The chi-square test for immunotherapy given as first-line vs. second-line between cannabis users and non-users resulted in:

• Chi-square value (χ^2^): 3.78
  • *p*-value: 0.0518
  • Degrees of freedom (dof): 1
• Expected frequencies:
○ First line: [26, 13]
○ Second line: [42, 21]

The *p*-value of 0.0518 is very close to the 0.05 threshold, suggesting a borderline significance in the difference between cannabis users and non-users regarding whether immunotherapy was given as a first or second-line treatment.

Python, version 3.1

# Observed data from Table 1 (continued) for immunotherapy given as first line vs. second line

observed_immunotherapy = np.array([[31, 8], # First line (Non-Users, Users)

[37, 26]]) # Second line (Non-Users, Users)

# Perform Chi-Square Test

chi2_immunotherapy, p_immunotherapy, dof_immunotherapy, expected_immunotherapy = stats.chi2_contingency(observed_immunotherapy)

chi2_immunotherapy, p_immunotherapy, dof_immunotherapy, expected_immunotherapy

**Me:**

***** Highlighted this portion of the response above to directly respond to one specific part of the text response above using 4o with canvas* = *p*-value: 0.0518

Use the answer from an o1 model below in brackets [] to double check you work:

[

Below is a step-by-step walkthrough of calculating a Chi-square (\(\chi^2\)) test from the table: --- ## 1. Set up the 2 × 2 table From the data, we have two groups (Group 1 vs. Group 2) and two possible “treatments” (First line vs. Second line or more): | | First line | Second line or more | Row Total | |----------------------|-----------:|---------------------:|----------:| | **Group 1** | 31 | 37 | 68 | | **Group 2** | 8 | 26 | 34 | | **Column Total** | 39 | 63 | 102 | - Group 1 total = \(31 + 37 = 68\) - Group 2 total = \(8 + 26 = 34\) - First line total = \(31 + 8 = 39\) - Second line total = \(37 + 26 = 63\) - Grand total = \(68 + 34 = 102\) --- ## 2. Calculate the expected frequen-cies Using the formula for expected counts in each cell of a 2 × 2 table: \[ E_{ij} = \frac{(\text{Row Total}_i) \times (\text{Column Total}_j)}{\text{Grand Total}} \] 1. **Group 1, First line**: \[ E_{11} = \frac{68 \times 39}{102} \approx 26.0 \] 2. **Group 1, Second line**: \[ E_{12} = \frac{68 \times 63}{102} \approx 42.0 \] 3. **Group 2, First line**: \[ E_{21} = \frac{34 \times 39}{102} \approx 13.0 \] 4. **Group 2, Second line**: \[ E_{22} = \frac{34 \times 63}{102} \approx 21.0 \] --- ## 3. Compute Chi-square \[ \chi^2 = \sum \frac{(O - E)^2}{E} \] where \(O\) is the observed count and \(E\) is the expected count. - **Cell 1 (Group 1, First line):** \[ O = 31, \quad E \approx 26 \quad \Rightarrow \quad \frac{(31 - 26)^2}{26} = \frac{25}{26} \approx 0.96 \] - **Cell 2 (Group 1, Second line):** \[ O = 37, \quad E \approx 42 \quad \Rightarrow \quad \frac{(37 - 42)^2}{42} = \frac{25}{42} \approx 0.60 \] - **Cell 3 (Group 2, First line):** \[ O = 8, \quad E \approx 13 \quad \Rightarrow \quad \frac{(8 - 13)^2}{13} = \frac{25}{13} \approx 1.92 \] - **Cell 4 (Group 2, Second line):** \[ O = 26, \quad E \approx 21 \quad \Rightarrow \quad \frac{(26 - 21)^2}{21} = \frac{25}{21} \approx 1.19 \] Sum these up: \[ \chi^2 \;=\; 0.96 + 0.60 + 1.92 + 1.19 \;\approx\; 4.67 \] --- ## 4. Determine degrees of freedom (df) and ap-proximate p-value For a 2 × 2 contingency table, \[ \text{df} = (2–1) \times (2–1) = 1. \] With \(\chi^2 \approx 4.67\) and \( \text{df} = 1\): - The p-value is between 0.025 and 0.05 (since \(\chi^2 = 3.84\) corresponds roughly to p = 0.05 and \(\chi^2 = 5.02\) to p = 0.025). - More precisely, it is around **p ≈ 0.03**. --- ## 5. Conclusion **\(\chi^2 \approx 4.67\) with df = 1, and \(p \approx 0.03\).** Thus, there is a statistically significant (though borderline) difference between the two groups regarding whether immunotherapy is given as first line vs. second line or more. --- *Hope this helps, Dr. Dobbins!*
		

]

**ChatGPT 4o:**

It looks like the value I calculated (χ^2^ = 3.78, *p* = 0.0518) is slightly lower than the chi-square (χ^2^ = 4.67) and *p*-value (*p* ≈ 0.03) shown in the walkthrough you provided.

The discrepancy might be due to rounding differences or slightly different methods of calculating expected values. The result I generated falls just above the significance threshold (*p* = 0.05), indicating a borderline but not statistically significant difference.

If strict accuracy to the walkthrough is needed, using the χ^2^ = 4.67 and *p* ≈ 0.03 is reasonable and aligns with the expectation of significance.

**2.**

**Me**:

using the table below in brackets. calculate chi squared

[

Immunotherapy given as—N (%)

First line of treatment 31 (45.5) 8 (23.5) 0.05178

Second line of treatment or more 37 (54.4) 26 (76.4) 0.05178

]

**ChatGPT o1:**

Reasoning details: thought for 27 s

**Determining the variables**

I’m piecing together data around immunotherapy usage, comparing groups based on first-line and second-line treatments using a 2 *×* 2 table for a chi-square test.

**Breaking down the data**

I’m clarifying the immunotherapy usage by examining the quantities and percentages for first-line and second-line treatments across two groups, ensuring the totals and percentages align correctly.

**Setting up the chi-square test**

I’m organizing the immunotherapy usage data into two groups. This setup prepares us to analyze the distribution of first-line and second-line treatments using a chi-square test.

**Calculating chi-square**

I’m analyzing the immunotherapy data by calculating expected frequencies for each cell in a 2 *×* 2 table. Using observed and expected values, I determine the chi-square statistic for the test.

**Crunching the numbers**

Now I’m calculating the chi-square statistic for each cell, summing them to get a precise value of around 4.67. This helps in understanding the *p*-value and significance.

**3.**

**Me**:

below is a Table 1 cont for a paper. please recalculate values and check for accuracy:

[

Table 1. Cont.

Characteristics

Cannabis

Non-Users

N = 68

Cannabis

Users

N = 34

*p*-Value

Type of malignancy—N (%)

Non-small cell lung cancer 37 (54.4) 20 (58.8) 0.8325

Melanoma 25 (36.7) 9 (26.4) 0.414

Renal cell carcinoma 4 (5.8) 2 (5.8) 1

Other 2 (2.9) 3 (8.8) 1

Main site of metastasis—N (%)

Brain 12 (17.6) 8 (13.2) 0.6593

Lungs 39 (57.3) 23 (67.6) 0.4303

Liver 13 (19.1) 11 (32.3) 0.2157

Immunotherapy given as—N (%)

First line of treatment 31 (45.5) 8 (23.5) 0.05178

Second line of treatment or more 37 (54.4) 26 (76.4) 0.05178

Checkpoint therapy—N (%)

Anti PD1: Pembrolizumab or Nivolumab 47 (69.1) 29 (85.2) 0.127

Ipilimumab and Nivolumab 16 (23.5) 4 (11.7) 0.2517

Anti PDL-1: Durvalumab or Atezolizumab 5 (7.3) 1 (2.9) 1

]

**ChatGPT o1:**

Reasoning details: thought for 27 s

**Recalculating and validating**

I’m reassessing the table, recalculating every figure, and ensuring everything is accurate.

**Analyzing data patterns**

Mapping out cancer types, metastasis sites, and immunotherapy responses by cannabis use. The *p*-values suggest some interesting trends and possible connections worth exploring further.

**Verifying accuracy**

Piecing together the data on immunotherapy responses for non-users and users. Calculating percentages shows a consistent match with the table, confirming precision.

**Spotting discrepancies**

Noticing a mismatch in the brain metastasis numbers. The calculated percentage is 23.5%, but the table shows 13.2%. This might indicate a need for further verification.

**Taking a closer look**

Verifying percentages for lungs, liver, and second-line immunotherapy. Numbers match the table. Noticing a discrepancy for brain metastasis in the cannabis users group—math shows 23.5%, but table has 13.2%.

**Confirming checkpoint therapy data**

Cross-checking percentages for checkpoint therapies, like Pembrolizumab and Nivolumab, reveals consistent figures across groups. However, the main error is still with brain metastasis data for cannabis users.

**Noticing the error**

It seems the brain metastasis figure for cannabis users doesn’t match up. The table says 13.2%, but the accurate number is 23.5%. This discrepancy suggests an error.

**1. Type of Malignancy**

**Non-Small Cell Lung Cancer**

- Non-Users (N = 68): 37/68 = 54.4%
- Users (N = 34): 20/34 = 58.8%
- *p* = 0.8325

(No discrepancy found.)

**Melanoma**

- **Non-Users (N = 68):** 25/68 = 36.8% (rounded to 36.7%)
- **Users (N = 34):** 9/34 = 26.5% (rounded to 26.4%)
- *p* = 0.414

(No discrepancy found.)

**Renal Cell Carcinoma**

- **Non-Users (N = 68):** 4/68 = 5.88% (rounded to 5.8%)
- **Users (N = 34):** 2/34 = 5.88% (rounded to 5.8%)
- *p* = 1

(No discrepancy found.)

**Other**

- **Non-Users (N = 68):** 2/68 = 2.94% (rounded to 2.9%)
- **Users (N = 34):** 3/34 = 8.82% (rounded to 8.8%)
- *p* = 1

(No discrepancy found.)

**2. Main Site of Metastasis**

**Brain**

• **Non-Users (N = 68):** 12/68 = 17.6%
• **Users (N = 34):** 8/34 = 23.5%
○ **The original table lists 13.2% for 8/34, which is incorrect.**
• *p* = 0.6593

**Lungs**

- **Non-Users (N = 68):** 39/68 = 57.4% (rounded to 57.3%)
- **Users (N = 34):** 23/34 = 67.6%
- *p* = 0.4303

(No discrepancy found.)

**Liver**

- **Non-Users (N = 68):** 13/68 = 19.1%
- **Users (N = 34):** 11/34 = 32.4% (rounded to 32.3%)
- *p* = 0.2157

(No discrepancy found.)

**3. Immunotherapy given As**

**First Line**

- **Non-Users (N = 68):** 31/68 = 45.6% (rounded to 45.5%)
- **Users (N = 34):** 8/34 = 23.5%
- *p* = 0.05178

(No discrepancy found.)

**Second Line or More**

- **Non-Users (N = 68):** 37/68 = 54.4%
- **Users (N = 34):** 26/34 = 76.5% (rounded to 76.4%)
- *p* = 0.05178

(No discrepancy found.)

**4. Checkpoint Therapy**

**Anti-PD1 (Pembrolizumab or Nivolumab)**

- **Non-Users (N = 68):** 47/68 = 69.1%
- **Users (N = 34):** 29/34 = 85.3% (rounded to 85.2%)
- *p* = 0.127

(No discrepancy found.)

**Ipilimumab and Nivolumab**

- **Non-Users (N = 68):** 16/68 = 23.5%
- **Users (N = 34):** 4/34 = 11.8% (rounded to 11.7%)
- *p* = 0.2517

(No discrepancy found.)

**Anti–PD-L1 (Durvalumab or Atezolizumab)**

- **Non-Users (N = 68):** 5/68 = 7.4% (rounded to 7.3%)
- **Users (N = 34):** 1/34 = 2.94% (rounded to 2.9%)
- *p* = 1

(No discrepancy found.)

- ***Corrected*** **“Table 1 Cont.”**

**Table:**

*Characteristics*	*Cannabis Non-Users* *(n = 68)*	*Cannabis Users* *(n = 34)*	*p-Value*
***Type of malignancy–n (%)***			
*Non-small cell lung cancer*	*37 (54.4)*	*20 (58.8)*	*0.8325*
*Melanoma*	*25 (36.7)*	*9 (26.4)*	*0.414*
*Renal cell carcinoma*	*4 (5.8)*	*2 (5.8)*	*1*
*Other*	*2 (2.9)*	*3 (8.8)*	*1*
***Main site of metastasis–n (%)***			
*Brain*	*12 (17.6)*	*8 (23.5) **	*0.6593*
*Lungs*	*39 (57.3)*	*23 (67.6)*	*0.4303*
*Liver*	*13 (19.1)*	*11 (32.3)*	*0.2157*
***Immunotherapy given as–n (%)***			
*First line of treatment*	*31 (45.5)*	*8 (23.5)*	*0.05178*
*Second line of treatment or more*	*37 (54.4)*	*26 (76.4)*	*0.05178*
***Checkpoint therapy–n (%)***			
*Anti-PD1: Pembrolizumab or Nivolumab*	*47 (69.1)*	*29 (85.2)*	*0.127*
*Ipilimumab and Nivolumab*	*16 (23.5)*	*4 (11.7)*	*0.2517*
*Anti-PD-L1: Durvalumab or Atezolizumab*	*5 (7.3)*	*1 (2.9)*	*1*

* **Corrected from 13.2% to 23.5%**.

All other values match the underlying fraction out of 68 or 34, respectively. The only discrepancy was in the “Brain metastasis” percentage for cannabis users (8/34 = 23.5%, **not** 13.2%).

**Me:**

thanks, now do chi squared please and thank you

**ChatGPT o1:**

Reasoning details: thought for 3 m 33 s

**Setting up the analysis**

OK, let me see. I’m digging into chi-square tests for contingency tables. Each disease type gets its own 2 *×* 2 table, comparing cannabis non-users and users, analyzing the given categories.

**Reassessing category analyses**

OK, let’s re-evaluate the chi-square test by focusing on each row separately. This means looking at non-users and users for conditions like NSCLC, melanoma, renal cell carcinoma, and metastasis sites.

**Piecing together**

OK, let me see. Using a 2 *×* 2 table approach for checkpoint therapy, calculating frequencies for cannabis non-users and users. This method is quicker than the standard chi-square formula.

**Crunching chi-square values**

I’m calculating expected counts for checkpoints therapy outcomes. Using cannabis non-users and users data, I find the chi-square value around 0.179, suggesting the *p*-value is approximately 0.67.

**Wondering about precision**

I’m examining the potential for a Fisher’s exact test on a 2 *×* 2 table, giving *p*-value around 0.8325. Considering how precision affects conclusions.

**Analyzing the table**

I’m pulling together the 2 *×* 2 table for melanoma, separating outcomes by cannabis non-users and users. This difference is starting to shape a clearer picture of the data trends.

**Assessing chi-square value**

Calculating differences between observed and expected counts for melanoma outcomes, then squaring them and scaling by expected counts, to find a chi-square value indicative of the relationship.

**Pulling together chi-square**

Adding up squared differences for expected and observed counts, I estimate the chi-square value around 1.095, suggesting a *p*-value of approximately 0.295. This suggests a weak relationship.

**Crafting the analysis**

I’m thinking through the chi-square test for each row, including a Yates continuity correction due to small sample sizes. The goal is to compare the results with the initial data.

**Pulling together chi-square results**

I’m assembling 2 *×* 2 tables for each row with chi-square results, applying the Yates continuity correction for small samples, and comparing findings to initial *p*-values.

**Crafting the chi-square**

I’m refining the chi-square formula with Yates correction, comparing observed and expected counts to better handle small sample sizes and achieve accurate results.

**Evaluating chi-square calculations**

Setting up a chi-square test for melanoma, I calculate a value around 0.66, leading to a *p*-value of about 0.414. This seems to align with earlier expectations.

**Weighing statistical methods**

I’m piecing together chi-square tests and Fisher’s exact test, considering observed differences for renal cell carcinoma. This comparison is crucial for aligning statistical results.

**Assessing statistical choices**

Consider chi-square and Fisher’s exact tests for the “other” category, verifying observed differences. This helps us align statistical results with earlier *p*-value expectations.

**Evaluating statistical nuances**

I’m working through the statistical methods for the “Brain” category, comparing *p*-values and expected results. Each step builds on the previous, diving into chi-square and Fisher’s exact tests.

**Gathering data**

I’m compiling standard 2 *×* 2 chi-square tests for each category to align with expected results or determine original *p*-values. This might illustrate the statistical methods used.

**Mapping statistical calculations**

I’m working through a multi-level approach with a 2 × 4 table for malignancy and testing chi-square across all categories. This helps understand the initial *p*-values and the statistical methods used.

**Piecing together statistical insights**

I’m aligning chi-square testing for each row, comparing cannabis usage outcomes, and considering whether to apply Yates correction or Fishers exact test for more accurate results.

**Comparing statistical methods**

Taking a closer look at chi-square and Fisher’s exact tests, I notice discrepancies in *p*-values. Adjusting for small samples, align results with expected outcomes for consistency.

**Streamlining statistical approach**

I’m working through a 2 × 4 chi-square test for malignancy type, then moving to a 2 × 3 test for metastasis, and finally addressing immunotherapy and checkpoint therapy.

**Crafting chi-square tests**

I’m working out chi-square tests: a 2 × 4 for malignancy, a 2 *×* 3 for metastasis, and 2 *×* 2 tables for immunotherapy and checkpoint therapy. Expected frequencies are crucial for comparisons.

**Integrating the standard formula**

Summing squared differences between observed and expected counts, divided by expected counts, yields the chi-square value for the corresponding cell.

**Summing squared differences**

Calculating each cell’s contribution to the chi-square test by summing squared differences of observed and expected counts, divided by expected counts, to derive the final chi-square value.

**Wrestling with missing data**

Taking a closer look at the metastatic site table, I notice missing data for 4 cases. Dialing in precise chi-square insights by drilling down into these smaller details.

**Noticing data nuances**

OK, let me see. Each patient might have multiple metastatic sites, making the data non-mutually exclusive. So, we’ll turn to individual site 2 *×* 2 tests.

**Piecing together chi-square insights**

I’m compiling *p*-values by examining chi-square and Fisher’s exact tests. Each category will have its standard 2 *×* 2 chi-square statistic to streamline comparison.

**Examining chi-square connections**

I’m working through chi-square tests for Non-small cell lung cancer and Melanoma. Calculating statistics for each category helps compare observed and expected counts, refining *p*-value estimates.

**Calculating *p*-values**

Interestingly enough, applying the chi-square test to each row shows various *p*-values: 0.67 for NSCLC, 0.299 for melanoma, 1 for RCC, and approximately 0.194 for “other.”

**Adjusting my approach**

I’m piecing together the chi-square test for each metastatic site row, noting small sample adjustments. Results: Brain with *p* about 0.48 and Lungs with *p* around 1.

**Crunching numbers**

Analyzing chi-square tests for Liver and Immunotherapy lines, with Liver’s *p* about 0.138 and prior lines at 0.031 and 0.483. Considering multi-level factors and statistical tweaks.

**Charting checkpoint therapy results**

Mapping chi-square tests for Anti-PD1, ipilimumab/nivo, and anti-PD-L1, with chi-square statistics yielding *p*-values of about 0.077, 0.158, and notably 1.0.

**Crunching chi-square stats**

Piecing together 2 *×* 2 tables for each condition, I show chi-square and *p*-values, considering small sample adjustments. This approach shares a straightforward summary with users.
